# Consistency between patients and families in recognizing cancer chemotherapy side effects: A questionnaire survey

**DOI:** 10.1002/cnr2.1451

**Published:** 2021-05-28

**Authors:** Fukuko Horio, Tokunori Ikeda, Yurimi Arake, Nodoka Kawashima, Erina Eto, Makoto Matsukura, Isao Fujii, Yuji Uchida

**Affiliations:** ^1^ Faculty of Pharmaceutical Sciences Sojo University Kumamoto Japan; ^2^ Kumamoto Regional Medical Center Kumamoto Japan; ^3^ Kumamoto‐Ashikita Medical Center for The Severely Disabled Kumamoto Japan; ^4^ Department of Pediatrics Arao Municipal Hospital Kumamoto Japan

**Keywords:** chemotherapy, communication, patients with cancer, quality of life, side effect

## Abstract

**Background:**

Although the side effects of cancer chemotherapy impair a patient's quality of life, family members' awareness of side effects may relieve patient anxiety and distress.

**Aim:**

We investigated whether patients and their families were consistent in recognizing the occurrence and severity of symptomatic side effects of chemotherapy treatment for cancer.

**Methods and results:**

This was a prospective observational study. We administered a questionnaire survey to patients and family members to assess the frequency of occurrence (1: never, 2: almost never, 3: sometimes, 4: frequently, 5: almost always, 6: unknown) and the degree of severity (1: mild, 2: moderate, 3: severe, 4: extremely severe, 5: unknown) of physical and psychological symptoms associated with cancer chemotherapy. Weighted Kappa and Cramer coefficients were used to assess consistency between the two groups. We surveyed 20 pairs of patients (5 men, 15 women) and their families (10 men, 10 women); 17 pairs lived together. The median age was 65.5 years (interquartile [IQR], 58.75, 69.25) for patients and 61.00 years (IQR, 47.25, 71.25) for family members. Of patients, 17 had solid cancer, and three had leukemia. Family members mostly recognized objectively visible symptoms such as hair loss and development of spots and keratinization. However, it was difficult for families to detect invisible subjective symptoms such as weakness, dysesthesia, depressed mood, and unarticulated anxiety.

**Conclusions:**

The results indicated that recognition of invisible subjective symptoms in patients undergoing chemotherapy was difficult even for family members. Therefore, a multidisciplinary approach in which various medical professionals actively communicate with both patients and families is important. Information sharing in collaboration with patients and families could increase understanding of the patient's condition and optimize patient care.

## 1 INTRODUCTION

The number of tumor‐bearing patients has increased worldwide in recent years. Globally, 18.1 million people had cancer and 9.6 million people died from the disease in 2018,^1^ and cancer deaths are predicted to double by 2040.^1^ A cancer statistics forecast for Japan reported a predicted number of cancer cases and deaths for 2019 of approximately 1 million and 380 000, respectively. Cancer is no longer a rare disease—indeed; it affects one in two Japanese people.^2^

Medical progress has resulted in the development of many treatments for cancer, and various types of treatment are now available.^3^, ^4^, ^5^, ^6^, ^7^ Consequently, the overall survival of patients with cancer has improved annually. However, cancer treatment, especially chemotherapy, causes various side effects, such as fatigue, nausea, vomiting, and hair loss.^8^, ^9^, ^10^ The willingness of patients to undergo cancer treatment declines according to the severity of chemotherapy side effects.^11^, ^12^ Although many therapeutic drugs have been used to mitigate the side effects of chemotherapy (and some drugs can reduce or eliminate side‐effect symptoms), chemotherapy inevitably causes symptoms such as pain. Therefore, to minimize distress, it is important for patients who undergo chemotherapy to receive support from individuals who can recognize side effects, understand the patient's suffering, and sympathize with their physical and psychological pain. Such individuals usually comprise medical personnel (pharmacists, nurses, or doctors) or the patient's family.

Pharmacists approach patients with cancer from the perspective of pharmaceutical care. Active intervention by pharmacists in the treatment process enables patients with cancer to better control side effects such as vomiting, and thus improves their quality of life (QOL) and treatment satisfaction.^13^, ^14^ Similarly, Bando, Onishi, and Imai reported that nursing support improves co‐occurring symptoms associated with cancer therapy and can give patients hope.^15^ Additionally, Kamijo and Miyamura found that spirituality had a considerable effect on patient QOL, demonstrating the importance of psychological evaluation and care for patients.^16^ Therefore, the support of interdisciplinary teams can improve patient QOL by approaching patient care from different perspectives.

Medical doctors tend to underestimate side effects experienced by patients.^17^, ^18^ Therefore, physicians may not fully understand the extent of side effects in patients receiving chemotherapy, leading to patient frustration and greater suffering. Because physicians play a central role in interdisciplinary teams, it is possible that their underestimation of side effects in patients receiving chemotherapy affects the choice of medical care, with serious effects on the physical and psychological condition of patients. Moreover, patients may become wary of chemotherapy, which makes it harder for them to continue cancer treatment.

To break this negative cycle arising from the underestimation of chemotherapy side effects, it is essential to accurately recognize side effects as soon as possible and to adequately convey patient information to medical personnel, including doctors. As family members are closest to the patient, they may play an important role in this process. Family support gives patients with cancer a sense of security and improves their QOL.^19^ Because families of patients with cancer understand and sympathize with patient experiences of side effects or difficulties associated with chemotherapy, family members can be instrumental in providing appropriate support to patients. However, to do this, families must be able to adequately recognize the occurrence and severity of side‐effect symptoms in patients receiving chemotherapy. However, it is unclear to what extent family members accurately recognize these symptoms.

Therefore, in this study, we investigated the degree of concordance between patients and their families in recognizing the occurrence and severity of various chemotherapy side‐effect symptoms. The findings may inform collaborations between medical personnel and families to support patients with cancer.

## 2 METHODS

### 2.1 Participants and methods

This was a prospective observational study. Questionnaire survey forms were distributed to participants in the Kumamoto Cancer Salon Network (http://www2.kuh.kumamoto-u.ac.jp/Canconsultation/salon/index.html) in Japan from August 2018 to October 2019. Participants comprised patients who had received cancer chemotherapy and their families. The salon personnel distributed the questionnaires and explained the purpose of the research. Importantly, the investigation was conducted independently for patients and families; the data were subsequently linked using a common key code. Although we obtained questionnaire responses from 23 patient‐family pairs, data for three pairs were excluded because the patients had not undergone chemotherapy. Finally, 20 patients and their families were enrolled.

### 2.2 Question items

The questionnaire items were based on the patient‐reported outcomes version of the Common Terminology Criteria for Adverse Events (PRO‐CTCAE).^20^ This is a measurement system developed by the National Cancer Institute to assess symptomatic adverse events experienced by patients in cancer clinical trials. As candidate questionnaire items, we selected those items from the PRO‐CTCAE that were consensually recognized by medical doctors, two nurses, and one pharmacist as associated with cancer treatment. To determine the appropriateness of the selected items, we conducted a preliminary questionnaire survey of 42 patients who had experienced cancer treatment. We asked about symptoms of cancer chemotherapy side effects using forced choice and open‐ended questionnaire items. Per the results of this preliminary survey, the questionnaire was modified and then completed after discussion and proofreading by the doctors, nurses, and pharmacist. At this stage, five patients who had undergone cancer chemotherapy were asked to fill in questionnaires to evaluate the understandability and validity of the questions. Subsequently, the questionnaire was revised slightly, and then finally administered to the participants of this study. The main part of the questionnaire comprised two questions for patients: (1) Please select the typical frequency of occurrence of chemotherapy‐associated symptoms. (2) Please select the typical degree of severity of chemotherapy‐associated symptoms. Family members were asked to respond to two similar questions: (1) Please select the typical frequency of occurrence of chemotherapy‐associated symptoms in the patient, according to your observations. (2) Please select the typical degree of severity of chemotherapy‐associated symptoms in the patient, according to your observations.

Chemotherapy‐associated symptoms comprised 19 items: fever, vertigo, weakness, stomatitis, dysgeusia, nausea, vomiting, constipation, diarrhea, anorexia, skin eruptions, spots and keratinization, edema, nail degeneration, dysesthesia, hair loss, depressed mood, and unarticulated anxiety. Ratings of the frequency of symptom occurrence were divided into six categories, and we constructed dummy variables corresponding to these categories (1: never, 2: almost never, 3: sometimes, 4: frequently, 5: almost always, 6: unknown). Ratings of the degree of severity were divided into five categories (1: mild, 2: moderate, 3: severe, 4: extremely severe, 5: unknown). We also asked about side‐effect symptoms that the patients found particularly troubling using an open‐ended question. Additionally, we collected information about type of cancer, cancer staging, and age at cancer diagnosis for each patient, and the relationship between the patient and their family. Data on sex and age for the two groups were collected at the time of the survey.

### 2.3 Statistical analysis

Descriptive statistics were used to evaluate the characteristics of each variable. To analyze differences in recognition of the occurrence and severity of each chemotherapy‐associated symptom in each patient‐family pair, we used weighted Kappa coefficients. Cramer's coefficient was used to confirm the association between chemotherapy‐related symptoms in patient and family groups. The statistical analyses were conducted using R software, version 3.6.2 (The R Foundation for Statistical Computing, Vienna, Austria). *Values of p* < .05 were considered significant.

## 3 RESULTS

### 3.1 Participant characteristics

We received questionnaire responses from 23 patients and their families (i.e., 23 patient‐family pairs). Of the 23 pairs, three were excluded because the patients had not been treated with cancer chemotherapy. The characteristics of the remaining 20 pairs are shown in Table 1. The median age was 65.50 years (interquartile range [IQR] 58.75, 69.25) in the patient group and 61.00 years (IQR 47.25, 71.25) in the family group. Three‐quarters of the patient group were women. Regarding type of cancer, 17 patients had solid cancer, and three had leukemia (Table 1, Table S1). A total of 10 patients were still undergoing cancer chemotherapy treatment (Table 1). More than half of patients responded “frequently” or “almost always” with respect to the occurrence of weakness, dysgeusia, constipation, nail degeneration, dysesthesia, hair loss, depressed mood, and unarticulated anxiety, and reported that these symptoms were severe (Table 2). Responses to the open‐ended question about the side‐effect symptoms that patients found particularly troubling showed the same pattern (data not shown).

**TABLE 1:** Participant characteristics

	Patient (*n* = 20)	Family (*n* = 20)
Age (y), median, IQR	65.50 (58.75, 69.25)	61.00 (47.25, 71.25)
Sex (male/female)	5/15	10/10
Age at cancer diagnosis	58.50 (54.25, 63.25)	NA
Type of cancer (solid/leukemia)	17/3	NA
Time duration between last chemotherapy and the assessment (on going/<1 year/<3 years/<5 years/<10 years/≥10 years)	10/1/1/4/4/0	NA
Parent/partner/offspring	NA	2/14/4
Cohabitation/Separation	17/3	17/3

Abbreviations: IQR, interquartile range; NA, not applicable.

**TABLE 2:** Survey response results

	Patient (*n* = 20)	Family (*n* = 20)
Occurrence of symptoms (never/almost never/sometimes/frequently/almost always/unknown), *n*		
Fever	4/3/9/2/2/0	4/2/8/2/2/2
Vertigo	6/5/6/2/1/0	6/5/3/2/0/3
Weakness	0/0/3/7/10/0	1/0/2/6/9/2
Stomatitis	7/2/9/0/2/0	6/2/4/3/1/3
Dysgeusia	3/6/1/1/9/0	2/4/2/3/5/3
Nausea	1/6/6/4/3/0	3/3/4/4/4/1
Vomiting	6/7/4/1/2/0	5/5/3/1/2/3
Constipation	1/6/2/4/6/1	2/1/4/2/6/5
Diarrhea	2/3/9/5/1/0	3/2/7/3/1/3
Anorexia	1/2/7/5/4/1	2/1/4/3/8/1
Skin eruption	5/4/3/2/3/0	3/8/3/2/1/2
Spots and keratinization	5/2/4/3/5/1	4/3/6/1/2/3
Edema	4/5/4/4/2/1	2/4/3/3/4/3
Skin peeling on limbs	7/6/4/0/3/0	4/5/5/1/0/4
Nail degeneration	1/2/3/4/9/0	1/7/2/3/5/1
Dysesthesia	1/3/4/3/8/1	1/1/5/3/6/3
Hair loss	1/3/2/2/9/2	1/4/0/3/11/0
Depressed mood	0/1/6/9/4/0	0/1/5/5/5/3
Unarticulated anxiety	0/2/3/6/7/2	0/2/3/2/8/4
Severity of symptoms (mild/moderate/severe/extremely severe/unknown), *n*		
Fever	6/6/5/2/0	3/5/2/4/6
Vertigo	6/6/5/1/0	8/3/2/1/5
Weakness	0/4/5/10/1	1/6/3/9/1
Stomatitis	7/7/3/3/0	8/5/0/3/3
Dysgeusia	6/5/2/7/0	4/2/4/5/4
Nausea	3/9/2/5/0	4/7/1/4/3
Vomiting	8/6/3/2/0	6/4/2/3/4
Constipation	3/5/4/7/0	4/3/4/3/6
Diarrhea	3/7/7/3/0	7/5/2/2/3
Anorexia	0/8/5/7/0	1/8/5/5/1
Skin eruption	8/4/4/3/0	5/9/2/1/3
Spots and keratinization	6/7/1/5/0	5/7/1/2/4
Edema	7/4/2/5/0	3/6/1/5/4
Skin peeling on limbs	12/1/3/3/1	6/7/1/0/5
Nail degeneration	2/4/5/7/1	8/3/5/2/1
Dysesthesia	3/3/4/7/1	5/2/2/7/4
Hair loss	3/3/0/11/1	8/1/0/10/1
Depressed mood	0/6/7/5/1	0/7/2/8/2
Unarticulated anxiety	0/4/6/8/1	0/5/2/8/5

Family members mostly responded “unknown” to questions about the occurrence and severity of the patients' symptoms (Table 2).

### 3.2 Agreement between patients and family

To evaluate the one‐to‐one correspondence between patient and family responses, we used weighted Kappa coefficients (Table 3). There was relatively high agreement between patient‐family pairs on recognition of the occurrence of the symptoms fever (0.49, *p* = .020), vertigo (0.45, *p* = .030), nausea (0.53, *p* = .015), anorexia (0.58, *p* = .008), spots and keratinization (0.74, *p* = .001), and hair loss (0.85, *p* < .001). However, there was low agreement for symptoms of weakness (0.06, *p* = .27), dysesthesia (−0.16, *p* = .48), depressed mood (0.13, *p* = .52), and unarticulated anxiety (0.20, *p* = .36).

**TABLE 3:** Weighted Kappa coefficients

	Occurrence of symptoms		Severity of symptoms	
	Coefficient	*p*‐value	Coefficient	*p*‐value
Fever	0.49	.020*	0.32	.046*
Vertigo	0.45	.030*	0.05	.80
Weakness	0.06	.27	−0.01	.96
Stomatitis	0.36	.07	0.06	.78
Dysgeusia	0.35	.12	0.23	.28
Nausea	0.53	.015*	0.32	.16
Vomiting	0.36	.07	0.28	.16
Constipation	0.24	.25	0.10	.63
Diarrhea	0.27	.18	0.049	.81
Anorexia	0.58	.008**	0.16	.47
Skin eruption	0.27	.28	0.33	.15
Spots and keratinization	0.74	.001**	0.49	.032*
Edema	0.35	.09	0.24	.30
Skin peeling on limbs	0.28	.17	0.56	.009**
Nail degeneration	0.30	.15	0.32	.05
Dysesthesia	−0.16	.48	0.03	.90
Hair loss	0.85	<.001***	0.68	.003**
Depressed mood	0.13	.52	−0.08	.72
Unarticulated anxiety	0.20	.36	0.19	.39

^*^ *p* < .05;

^**^ *p* < .01;

^***^ *p* < .001.

Regarding symptom severity, there was high agreement for spots and keratinization (0.49, *p* = .032), skin peeling on limbs (0.56, *p* = .009), and hair loss (0.68, *p* = .003). However, there were large discrepancies for vertigo (0.05, *p* = .80), weakness (−0.01, *p* = .96), stomatitis (0.06, *p* = .78), constipation (0.10, *p* = .63), diarrhea (0.049, *p* = .81), anorexia (0.16, *p* = .47), dysesthesia (0.03, *p* = .90), depressed mood (−0.08, *p* = .72), and unarticulated anxiety (0.19, *p* = .39). To confirm these results, we performed bootstrap analysis (*n* = 2000) of the weighted Kappa coefficients. The results showed a similar pattern of agreement (Table S2).

### 3.3 Association between question items

To evaluate the consistency between each question item, we calculated Cramer coefficients. Table 4 shows the relationship between items for which the coefficients were above 0.80. In the patient group, there was a high correlation between the occurrence and severity of hair loss (0.86) and between the occurrence and severity of dysesthesia (0.81). In the family group, there was a high correlation between the occurrence and severity of unarticulated anxiety (0.83), between the occurrence and severity of stomatitis (0.82), and between the occurrence and severity of constipation (0.80). Moreover, patients, but not families, showed high correlations (in occurrence or severity) between some physical and emotional symptoms, such as dysesthesia and unarticulated anxiety (0.91) and edema and depressed mood (0.81).

**TABLE 4:** Cramer coefficients ≥0.80 between question items

	Question item	Question item	Coefficient
Patient			
	Severity of dysesthesia	Severity of unarticulated anxiety	0.91
	Occurrence of hair loss	Severity of hair loss	0.86
	Occurrence of feebleness	Occurrence of hair loss	0.81
	Occurrence of edema	Severity of depressed mood	0.81
	Occurrence of dysesthesia	Severity of dysesthesia	0.81
Family			
	Severity of spots and keratinization	Severity of skin peeling on limbs	0.92
	Occurrence of dysesthesia	Occurrence of hair loss	0.86
	Occurrence of unarticulated anxiety	Severity of unarticulated anxiety	0.83
	Occurrence of stomatitis	Severity of stomatitis	0.82
	Occurrence of constipation	Severity of constipation	0.80

## 4 DISCUSSION

Assuming that participants would have different types of cancer, in this study, we focused on chemotherapy side effects common to many cancers. This helped to minimize the effect of the type of anticancer medicine on questionnaire responses. The results showed that patients frequently experienced symptoms such as weakness, dysgeusia, nail degeneration, hair loss, depressed mood, and unarticulated anxiety during cancer chemotherapy. They also rated the above symptoms as severe. We investigated the consistency in the recognition of side effects between patients receiving cancer chemotherapy and their families.

Of objectively visible symptoms, hair loss and development of spots and keratinization are well known and clearly visible side effects of general cancer chemotherapy.^8^, ^21^, ^22^, ^23^, ^24^, ^25^, ^26^, ^27^ Analysis of the one‐to‐one correspondence between patients and families in the recognition of specific symptom items showed a high degree of agreement. These results suggest that family members can recognize these symptoms in patients. However, the identity of family members may affect this consistency. For example, compared with offspring, partners usually spend more time with the patient and therefore may more readily notice the occurrence and severity of the patient's symptoms. However, most of our patient participants lived with several relatives, so we consider the effect of family member identity to be small.

Regarding the recognition of other symptoms in patients with cancer, Mulders, Vingerhoets, and Breed reported that perceptions about the effects of cancer and chemotherapy among medical doctors and nurses were approximately the same, but the perceptions of health professionals and patients differed.^28^ Basch and Kawaguchi et al. found that physicians tended to underestimate patient side effects.^17^, ^18^ Sakai, Umeda, Okuyama, and Nakamura reported a high concordance in perceptions of side effects between nurses and patients but a low concordance between doctors and patients.^29^ Thus, physicians and patients may differ in their recognition of side effects. If families are aware of patient side effects, they can act as a bridge between patients and medical professionals such as doctors and nurses. Therefore, it is important that family members accurately recognize side effects in patients. Family reports can increase medical professionals' awareness of side effects, making it possible to more accurately assess the patient's condition and take measures appropriate to their symptoms. Therefore, it is important for health care professionals to communicate with both patients and families to obtain more accurate information and take appropriate action.

Receiving additional information about patients could lead to improved patient care by medical personnel. Mulders et al. reported a large discrepancy between nurses and doctors in the recognition of symptoms in patients with cancer.^28^ If medical professionals shared family reported patient information, they could provide optimal support for the patient's symptoms. Hence, the presence of family and their information about the patient can serve as a bridge to and among medical professionals such as doctors, nurses, and pharmacists.

Invisible subjective symptoms such as weakness, dysesthesia, depressed mood, and unarticulated anxiety are frequently occurring side effects.^9^, ^11^, ^30^ Our results showed that it was difficult for family members to recognize these symptoms in patients. The severity of dysesthesia was strongly associated with psychological side effects in the patient group but not in the family group. Physical side effects may affect the patient's experience of psychological side effects.^31^, ^32^ Therefore, if severe invisible clinical symptoms such as dysesthesia are not adequately noticed and not appropriately treated, the patient may experience worse psychological symptoms. To avoid this, the input of family is important, because they are usually closest to the patient. Family members can potentially understand the patient's condition and recognize invisible, subjective symptoms by discussing with the patient feelings such as anxiety that cannot be easily conveyed in other ways.

Many patients with cancer are worried about the effect of their illness on their family.^33^ Moreover, some studies have reported that the families of patients with cancer may experience emotional distress and depression.^34^, ^35^ Therefore, patients may avoid telling their families about invisible subjective symptoms like mental conflict or anxiety; the differences found here between patients and their families in recognizing invisible subjective symptoms may reflect this.

Medical professionals can help to resolve problems arising from unrecognized patient symptoms. Health care professionals must provide appropriate information to help patients to accurately understand their condition, such as the selected treatment strategy, type, and frequency of side effects, and how to control anxiety as much as possible. Additionally, when health professionals explain side‐effect symptoms to the patient's family, it is important that they mention not only visible symptoms but also the possible occurrence of invisible symptoms. It may be difficult for families to understand a single explanation, so medical personnel should repeatedly and carefully explain the relevant information. Furthermore, although doctors play a central role in providing information to patients and families, intervention by other medical professionals, such as pharmacists and nurses, is also needed. Patients sometimes hesitate to communicate with their families and physicians^36^, ^37^; therefore, pharmacists and nurses should try to share more information with patients about side effects and how to cope with worry or anxiety. In this way, pharmacists and nurses could serve as a bridge between medical personnel, patients, and families, and could help families to become aware of invisible side effects and their severity at an early stage. This process would enable health care professionals to support both patients and their families.

This study has some limitations. First, although we conducted a pilot study to increase the reliability of the findings, and used a bootstrap method to increase statistical robustness, the sample size was small. Second, participants were recruited only through the Kumamoto Cancer Salon. Owing to the limited target sampling area, the relationships between patients and their families may have been affected by local characteristics. Third, because the number of chemotherapy sessions affects the occurrence and severity of side effects, the families of patients who had received many chemotherapy sessions may have been more likely to notice chemotherapy‐associated changes in the patient. However, we did not record the number of chemotherapy sessions in this study. Fourth, about half of patients were still undergoing treatment during the questionnaire survey. The families of patients with ongoing treatment may more easily have recognized the occurrence and severity of side effects than families of patients who had completed chemotherapy. The time from the last chemotherapy to this survey may also have affected the results. Therefore, our findings may have been subject to recall bias.

Additional research is necessary to expand the study area and further investigate the reliability and validity of these data. Here, we investigated the perceptions of patients and their families regarding side effects of cancer chemotherapy. Few studies have examined the one‐to‐one correspondence between patient and family reports of chemotherapy side effects. The findings could inform strategies for appropriate medical care in patients undergoing chemotherapy.

## 5 CONCLUSIONS

The side effects of cancer chemotherapy greatly affect patient QOL. In this study, families and patients tended to agree on their perceptions of objective side‐effect symptoms, but agreement was lower for subjective side‐effect symptoms. Among patients, there was a strong relationship between the occurrence or severity of physical symptoms and psychological symptoms. To fully understand the patient's physical and psychological condition, ongoing communication is very important (e.g., from patients to doctors, patients to nurses, patients to pharmacists, patients to families, families to medical personnel, and medical personnel with each other). Furthermore, because symptoms, feelings, and ways of thinking about side effects differ among patients, interventions tailored to each patient are necessary. Therefore, it is important for medical professionals to cooperate as a team; for example, by obtaining appropriate information through better communication, sharing the obtained information, and subsequently supporting the patient from different perspectives. Multidisciplinary care by various specialists may improve patient QOL. This would support patients and their families by alleviating anxiety, generating hope, and actively engaging patients and families in treatment. Finally, such cooperation could make cancer treatment easier for patients and facilitate positive outcomes.

## CONFLICT OF INTEREST

The authors have stated explicitly that there are no conflicts of interest in connection with this article.

## AUTHORS' CONTRIBUTIONS

All authors had full access to the data in the study and take responsibility for the integrity of the data and the accuracy of the data analysis. *Conceptualization*, I.F., Y.U.; *Methodology*, F.H., Y.A., N.K., E.E., M.M., I.F.; *Investigation*, F.H., Y.A., N.K.; *Formal Analysis*, T.I.; *Data Curation*, T.I., Y.A., N.K.; *Writing ‐ Original Draft*, F.H., T.I.; *Writing ‐ Review & Editing*, T.I., Y.A., N.K., E.E., M.M., I.F., Y.U.; *Supervision*, I.F., Y.U.

## ETHICAL STATEMENT

This research was approved by the research ethics committee of Sojo University Faculty of Pharmacy (number: 2019‐1, approval date: June 3, 2019). Written informed consent was obtained from all participants after they had received a full explanation of the procedure on the questionnaire forms. The information from all surveys was kept anonymous.

## Supporting information


**Data S1.** Supporting information.

## Data Availability

The data that support the findings of this study are available from the corresponding author upon reasonable request.
