# Supplementary material for: Consistency between patients and families in recognizing cancer chemotherapy side effects: A questionnaire survey
Source: Cancer Rep (Hoboken). 2021 May 28;5(1):e1451. doi: 10.1002/cnr2.1451 (PMC8789603; doi:10.1002/cnr2.1451)
Supplement: Supplementary file 1 — Data S1. Supporting information. [file CNR2-5-e1451-s001.docx]

**Supplemental Table 1. Cancer type of participant patients**

| **Solid** | **Leukemia** |
| --- | --- |
| Colorectal cancer | Malignant lymphoma |
| Breast cancer |  |
| Cancer of uterine body |  |
| Lung cancer |  |
| Ovarian cancer |  |
| Pancreatic cancer |  |
| Bile duct cancer |  |
| Prostate cancer |  |
| Stomach cancer |  |

**Supplemental Table 2. Weighted Kappa coefficients (N = 2,000 bootstrap replications)**

|  | **Occurrence of symptoms** | | **Severity of symptoms** | |
| --- | --- | --- | --- | --- |
|  | Coefficient | P-value | Coefficient | P-value |
| Fever | 0.51 | 0.014* | 0.41 | 0.006** |
| Vertigo | 0.58 | 0.005** | 0.08 | 0.72 |
| Weakness | -0.29 | 0.12 | 0.07 | 0.75 |
| Stomatitis | 0.21 | 0.17 | 0.01 | 0.97 |
| Dysgeusia | 0.36 | 0.09 | 0.39 | 0.08 |
| Nausea | 0.84 | < 0.001*** | 0.47 | 0.028* |
| Vomiting | 0.56 | 0.016* | 0.11 | 0.50 |
| Constipation | -0.03 | 0.86 | 0.41 | 0.06 |
| Diarrhea | 0.21 | 0.30 | 0.29 | 0.16 |
| Anorexia | 0.55 | 0.014* | 0.38 | 0.07 |
| Skin eruption | -0.12 | 0.53 | 0.40 | 0.08 |
| Spot and keratinization | 0.65 | 0.006** | 0.52 | 0.018* |
| Edema | 0.44 | 0.06 | 0.33 | 0.09 |
| Skin peeling of limbs | 0.04 | 0.83 | 0.71 | < 0.001*** |
| Nail degeneration | 00.06 | 0.78 | 0.49 | 0.018* |
| Dysesthesia | -0.16 | 0.48 | 0.24 | 0.24 |
| Hair loss | 0.81 | < 0.001*** | 0.70 | < 0.001*** |
| Depressed mood | 0.22 | 0.24 | -0.06 | 0.76 |
| Unarticulated anxiety | 0.13 | 0.54 | 0.25 | 0.25 |

**p* < 0.05.

***p* < 0.01.

****p* < 0.001.
